# Antineutrophil Cytoplasmic Antibody-Associated Vasculitis in a Pediatric Patient Presenting With Hemoptysis

**DOI:** 10.7759/cureus.68631

**Published:** 2024-09-04

**Authors:** Ashley Anderson, Brian Bartlett, James Chally, Joseph Malicki

**Affiliations:** 1 Emergency Medicine, Mayo Clinic Health System, Mankato, USA

**Keywords:** polyarthralgia, hemoptysis, subglottic stenosis, pediatric granulomatosis with polyangiitis, granulomatosis with polyangiitis, anca

## Abstract

This case report discusses a rare pediatric case of granulomatosis with polyangiitis (GPA) presenting with hemoptysis, migratory polyarthralgia, significant laboratory abnormalities, and imaging findings.

GPA is a form of vasculitis that primarily affects the upper and lower respiratory tracts and the kidneys. Pediatric cases, though rare, offer a distinct set of clinical challenges.

The patient presented to the emergency department with hemoptysis, joint pain, and cough. Radiologic findings included diffuse bilateral nodular airspace opacities on chest X-ray (CXR) and ground glass opacities on computed tomography (CT). After hospital admission, the patient's bronchoscopy suggested diffuse alveolar hemorrhage. Laboratory tests were positive for proteinase 3 (PR3), indicating a possible diagnosis of GPA. Further tests, consultations, and evaluations corroborated this diagnosis. Treatment administered included high-dose intravenous steroids, rituximab, and other supportive measures.

Pediatric GPA, while rare, is a challenging diagnostic entity. A comprehensive and multidisciplinary approach is pivotal for timely diagnosis and initiation of appropriate therapy.

## Introduction

Antineutrophil cytoplasmic antibody (ANCA)-associated vasculitis encompasses a group of diseases characterized by inflammation of small to medium-sized blood vessels, including granulomatosis with polyangiitis (GPA). GPA, formerly known as Wegener's granulomatosis, primarily affects the upper and lower respiratory tracts and the kidneys​ [1]. While GPA is more commonly observed in adults, pediatric onset is rare and presents unique clinical challenges due to differences in disease presentation and progression [2]. Pediatric patients often present with symptoms such as subglottic stenosis and hemoptysis, whereas adults are more likely to present with epistaxis and sinusitis. Children are also more likely to exhibit symptoms of arthralgia and myalgia [3].

Existing literature on pediatric GPA is limited, and most studies focus on adult populations, leading to a gap in knowledge regarding the diagnosis and management of this disease in children. Previous studies have indicated that pediatric patients with GPA may experience more severe disease courses and complications compared to adults [4]. This case report aims to fill this gap by detailing a rare presentation of pediatric GPA contributing to the overall understanding and care of pediatric patients with ANCA-associated vasculitis.

This case report also underscores the importance of considering GPA in the differential diagnosis when pediatric patients present with persistent respiratory symptoms, including hemoptysis, with arthralgia and myalgia. While viral illnesses are a more common etiology for myalgia and arthralgia, the presence of hemoptysis should heighten clinical concern for ANCA-associated vasculitis. Early recognition and consideration of GPA are crucial for timely and appropriate treatment, thereby improving patient outcomes. This case serves as a valuable addition to the existing body of knowledge, emphasizing the need for heightened awareness and comprehensive care strategies among healthcare providers [5].

## Case presentation

**Figure 1:**
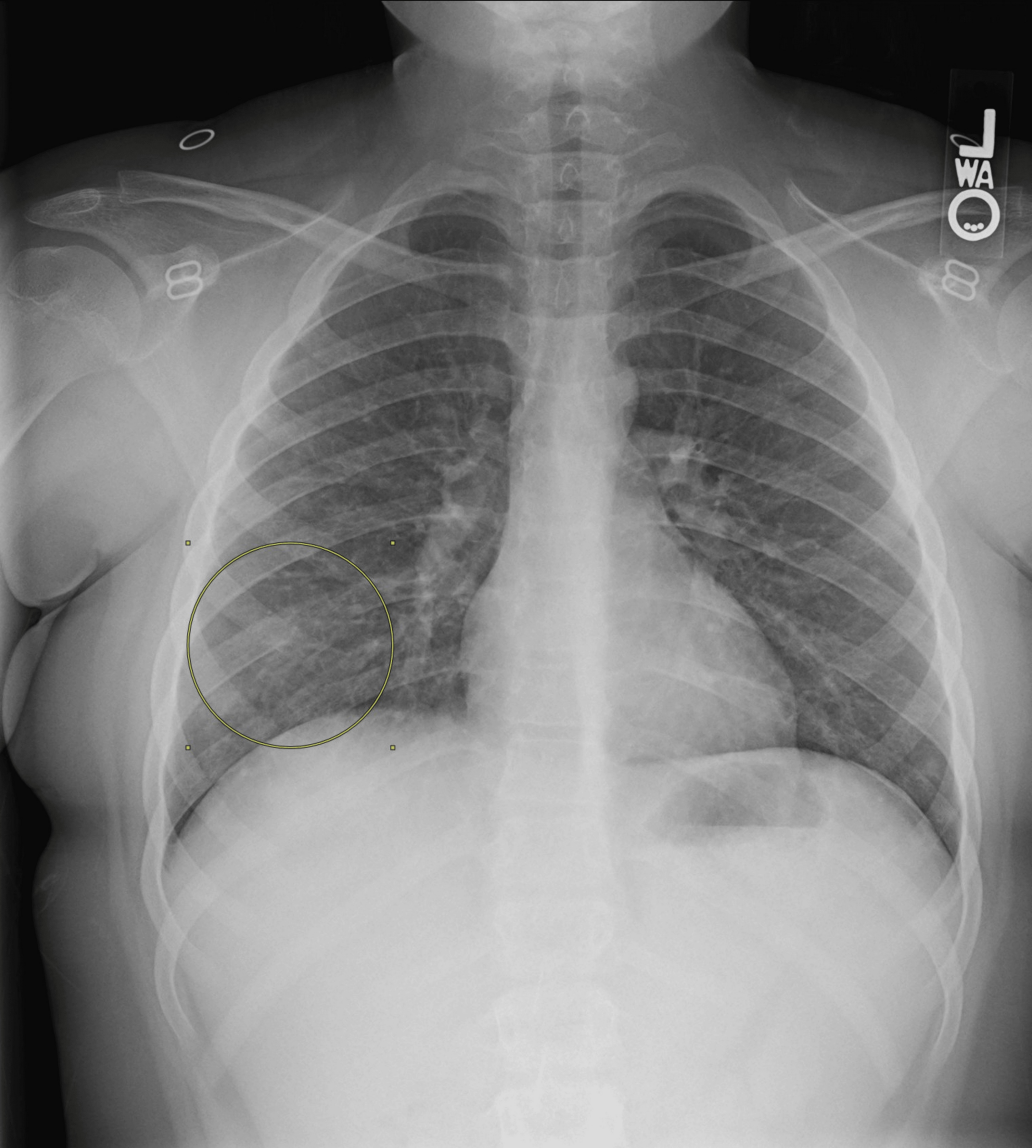
Initial chest X-ray during urgent care evaluation

A nine-year-old otherwise healthy female presented to a regional emergency department with a persistent cough for the past two weeks and two episodes of hemoptysis on the day prior to evaluation. She also described pain in her right wrist, both ankles, elbows, and right second through fifth fingers over the last three days but denied swelling. Her parents reported that she had no prior medical history. However, she was seen six days before the emergency visit at an urgent care center for a week-long cough, fatigue, and three episodes of hemoptysis. The urgent care provider prescribed 250 mg of oral (PO) cefdinir after a chest X-ray (CXR) indicated possible pneumonia (Figure 1). Despite the antibiotic treatment, her symptoms persisted throughout the week, and she experienced two additional episodes of hemoptysis the day before her emergency department presentation. The parents described the child expectorating fresh blood and blood clots, providing photos of the expectorant that showed golf ball-sized amounts of blood per incident.

On physical examination, the patient was tachypneic with a respiratory rate of 26 breaths per minute, but no use of accessory muscles, tracheal tugging, nasal flaring, or paradoxical breathing was observed. She had a heart rate of 122 beats per minute, oxygen saturation of 94% on room air, blood pressure of 110/71 mmHg, and temporal temperature of 37.1°C. Lung sounds were slightly diminished, with no wheezing, rales, or rhonchi. A persistent cough was observed, occurring one to two times per minute, which did not appear to cause significant distress. She had no abdominal pain and skin examination was normal. She experienced pain with flexion and extension of her right wrist and the second through fifth fingers, but no swelling, fluctuance, erythema, or external lesions were observed in these joints. Otherwise, her ankles, knees, hips, elbows, left wrist, shoulders, and other fingers were without pain during range of motion, and had no swelling, fluctuance, erythema, or lesions.

**Figure 2:**
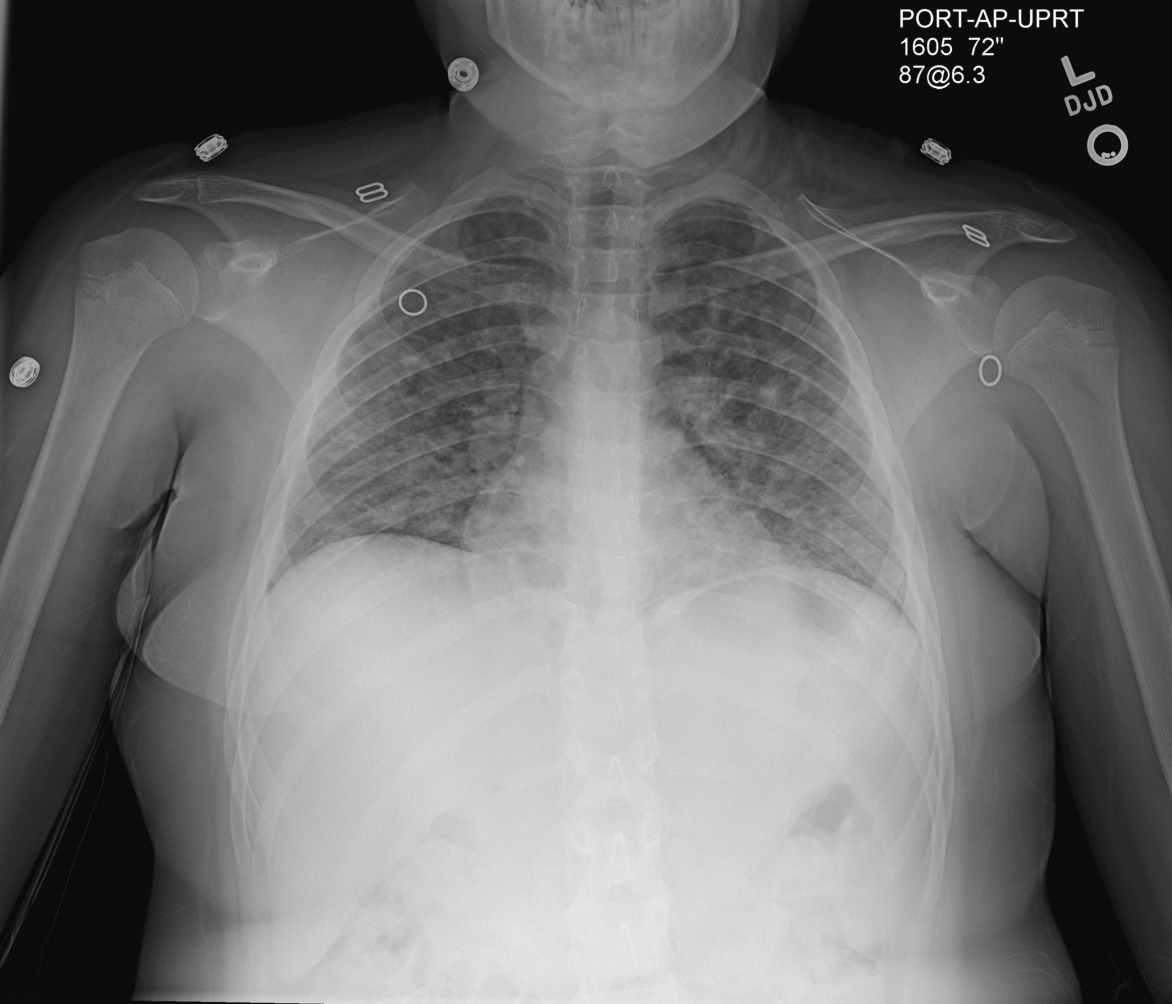
Emergency department chest X-ray

Initial one-view CXR demonstrated diffuse bilateral nodular airspace opacities of infectious or inflammatory etiology (Figure 2). These findings had developed since her CXR at urgent care six days prior.

Relevant laboratory results included anemia (hemoglobin 10.1 g/dL), hypokalemia (3.2 mmol/L), hypocalcemia (8.7 mg/dL), normal creatinine (0.69 mg/dL), and an elevated C-reactive protein (CRP) of 36.0 mg/L (Table 1). Given her clinical history, hemoptysis, and CXR findings, there was a suspicion of alveolar hemorrhage of uncertain etiology. Pediatric Pulmonology was consulted and recommended that the child be transferred to their facility for emergent bronchoscopy.

**Table 1:** Chronological laboratory investigations and reference ranges HPF: high-power field

Test	Initial value	Follow-up value	Reference range
Hemoglobin (g/dL)	10.1 g/dL	11.5 g/dL	12.0 - 15.5 g/dL
Potassium (mmol/L)	3.2 mmol/L	-	3.5 - 5.1 mmol/L
Calcium (mg/dL)	8.7 mg/dL	-	8.6 - 10.3 mg/dL
Creatinine (mg/dL)	0.69 mg/dL	0.66 mg/dL	0.6 - 1.2 mg/dL
C-reactive protein (CRP) (mg/L)	36.0 mg/L	-	< 10 mg/L
Urine protein (mg/dL)	500 mg/dL	0 mg/dL	0 - 8 mg/dL
Red blood cells (RBCs) in urine (per HPF)	-	51-100 per HPF	0 - 5 per HPF
Cystatin C (mg/L)	-	1.72 mg/L	0.52 - 0.98 mg/L

At the accepting hospital, the child underwent bronchoscopy, which revealed diffuse alveolar hemorrhage in all lung fields. Laboratory evaluation of secretions was positive for proteinase 3 (PR3) antibodies, which have a specificity of approximately 90-95% and a sensitivity of 70-90% for GPA, making it a significant marker in diagnosing ANCA-associated vasculitis [6]. She was formally diagnosed with GPA based on the presence of PR3 antibodies, clinical features of systemic vasculitis including alveolar hemorrhage, and histopathological evidence from the kidney biopsy showing necrotizing and crescentic glomerulonephritis with paucity of immune deposits. She was started on 500 mg of intravenous (IV) methylprednisolone twice daily and a single dose of 1000 mg of IV rituximab. Renal ultrasound showed Society for Fetal Urology (SFU) grade 1 pelviectasis, and subsequent kidney biopsy revealed necrotizing and crescentic glomerulonephritis with a paucity of immune deposits.

During her hospital stay, the child showed progressive improvement with immunotherapy. She experienced one episode of hypertension with a blood pressure of 164/96 mmHg after receiving 500 mg of IV methylprednisolone, which required a single dose of 5 mg of oral (PO) isradipine. Due to her immunosuppressive regimen, she was started on a prophylactic dose of 800 mg of oral sulfamethoxazole and 160 mg of trimethoprim three times weekly. She was also started on 1000 units of PO vitamin D and 1000 mg of calcium supplements daily to counteract the potential bone density loss associated with long-term corticosteroid use. She was successfully transitioned to 60 mg of PO prednisone and discharged on day six of hospitalization. She was referred to Ophthalmology for monitoring of potential retinal vasculitis, and a repeat dose of 1000 mg of IV rituximab was recommended six months after the initial dose. Discharge medications included prednisone 60 mg PO daily for four weeks, pantoprazole 40 mg PO daily while on steroids, and sulfamethoxazole 800 mg and trimethoprim 160 mg PO to be taken three times weekly. Additionally, PO discharge medications included calcium carbonate 400 mg daily, vitamin D 1000 units daily, amlodipine 5 mg daily, and isradipine 2.5 mg as needed for blood pressure exceeding 135/90 mmHg.

During follow-up with her primary pediatrician one week after hospital discharge, the patient’s extra-renal disease appeared well controlled. The symptoms of cough, hemoptysis, and fatigue had completely resolved. Compared to her initial evaluation in the emergency department, her hemoglobin increased from 10.1 g/dL to 11.5 g/dL. The urine protein decreased from 500 mg/dL to 0 mg/dL, though she still had hematuria with 51-100 RBCs per high-power field. Serum creatinine decreased from 0.95 mg/dL to 0.66 mg/dL. Cystatin C was measured to provide an additional assessment of kidney function, yielding a result of 1.72 mg/L The patient was continued on all discharge medications, with a plan for monthly reassessments or as symptoms developed to determine optimal medication management.

## Discussion

This case report highlights the complex diagnostic journey of a nine-year-old female initially presenting with a persistent cough and hemoptysis. Initially managed as pneumonia at an urgent care visit, she was started on antibiotics based on a chest X-ray that suggested possible pneumonia. Despite this treatment, her symptoms persisted, leading to significant episodes of hemoptysis which prompted her visit to the emergency department.

A key takeaway from this case is the necessity of including ANCA-associated vasculitis in the differential diagnosis when pediatric patients present with persistent respiratory symptoms such as cough, hemoptysis, dyspnea, and constitutional symptoms like fatigue and weight loss. Neglecting to consider ANCA-associated vasculitis can result in delayed or missed diagnoses, potentially leading to severe outcomes such as irreversible renal failure, life-threatening pulmonary hemorrhage, and increased long-term morbidity. Pediatric GPA often presents with more severe symptoms, such as respiratory distress and renal involvement, and can lead to significant complications like pulmonary hemorrhage and renal failure if not promptly diagnosed and treated [6]. Comprehensive evaluation and early specialist involvement were crucial for managing and improving her prognosis [7]. Early recognition of GPA and prompt initiation of treatment with high-dose intravenous steroids and rituximab led to significant clinical improvement and prevented the progression of extra-renal disease, as demonstrated by the patient's rapid improvement and stabilization of symptoms [8,9]. Prior studies have demonstrated that multidisciplinary management, including early involvement of subspecialists, leads to improved outcomes in pediatric vasculitis cases [10]. 

The use of accurate and timely diagnostic tools, such as PR3 antibody testing and advanced imaging modalities like bronchoscopy, often available at tertiary referral centers, plays a critical role in the early identification of GPA. Referral to such centers for these advanced diagnostic tests can significantly aid in the early diagnosis and management of the disease. Recent advances in diagnostic techniques have significantly improved the sensitivity and specificity of ANCA testing, aiding in the prompt diagnosis and management of vasculitis [11]. This case highlights the importance of combining clinical evaluation with advanced diagnostic tools to achieve accurate and timely diagnosis.

This case report is unique due to its initial presentation and diagnostic challenges. Initially, the patient was diagnosed with pneumonia based on isolated chest X-ray findings at an urgent care visit, which is more suggestive of bacterial pneumonia rather than GPA. Only after this initial visit did she develop migratory arthritis and diffuse X-ray findings, which are more characteristic of GPA. Unlike typical cases of GPA, this patient lacked many characteristic features early on, making recognition more difficult. However, the case underscores the critical importance of early recognition and a multidisciplinary approach in managing pediatric vasculitis. A study by Little et al. demonstrated that early diagnosis and treatment significantly reduce morbidity and mortality in pediatric vasculitis [12]. Furthermore, research by Hellmich et al. supports the use of standardized diagnostic criteria and treatment protocols to improve patient outcomes [13]. This case highlights the need for heightened clinical suspicion and comprehensive management strategies in atypical presentations of GPA, emphasizing its uniqueness and value to the medical community.

## Conclusions

Granulomatosis with polyangiitis (GPA) in the pediatric population represents a diagnostic challenge due to its rarity and broad spectrum of presenting symptoms, which often mimic common viral illnesses. This case demonstrates the necessity of a comprehensive, multidisciplinary approach for timely diagnosis and initiation of therapy. The early involvement of specialists facilitated targeted investigations and rapid improvement in this patient's symptoms. Literature suggests that timely diagnosis and treatment of GPA lead to better clinical outcomes and can prevent severe complications.

This case also highlights the potential pitfall of misdiagnosing pneumonia in patients with pulmonary involvement of GPA. Initial treatment for suspected pneumonia delayed the correct diagnosis, underscoring the importance of considering GPA in the differential diagnosis for pediatric patients presenting with persistent cough, arthralgias, fatigue, and hemoptysis. Emergency clinicians should consider GPA in pediatric patients with these symptoms to expedite appropriate workup and care, thereby improving patient outcomes.
